# Supplementary material for: Muscle functions as a connective tissue and source of extracellular matrix in planarians
Source: Nat Commun. 2019 Apr 8;10:1592. doi: 10.1038/s41467-019-09539-6 (PMC6453901; doi:10.1038/s41467-019-09539-6)
Supplement: Supplementary file 1 — Supplementary Information [file 41467_2019_9539_MOESM1_ESM.pdf]

Supplementary Figure 1

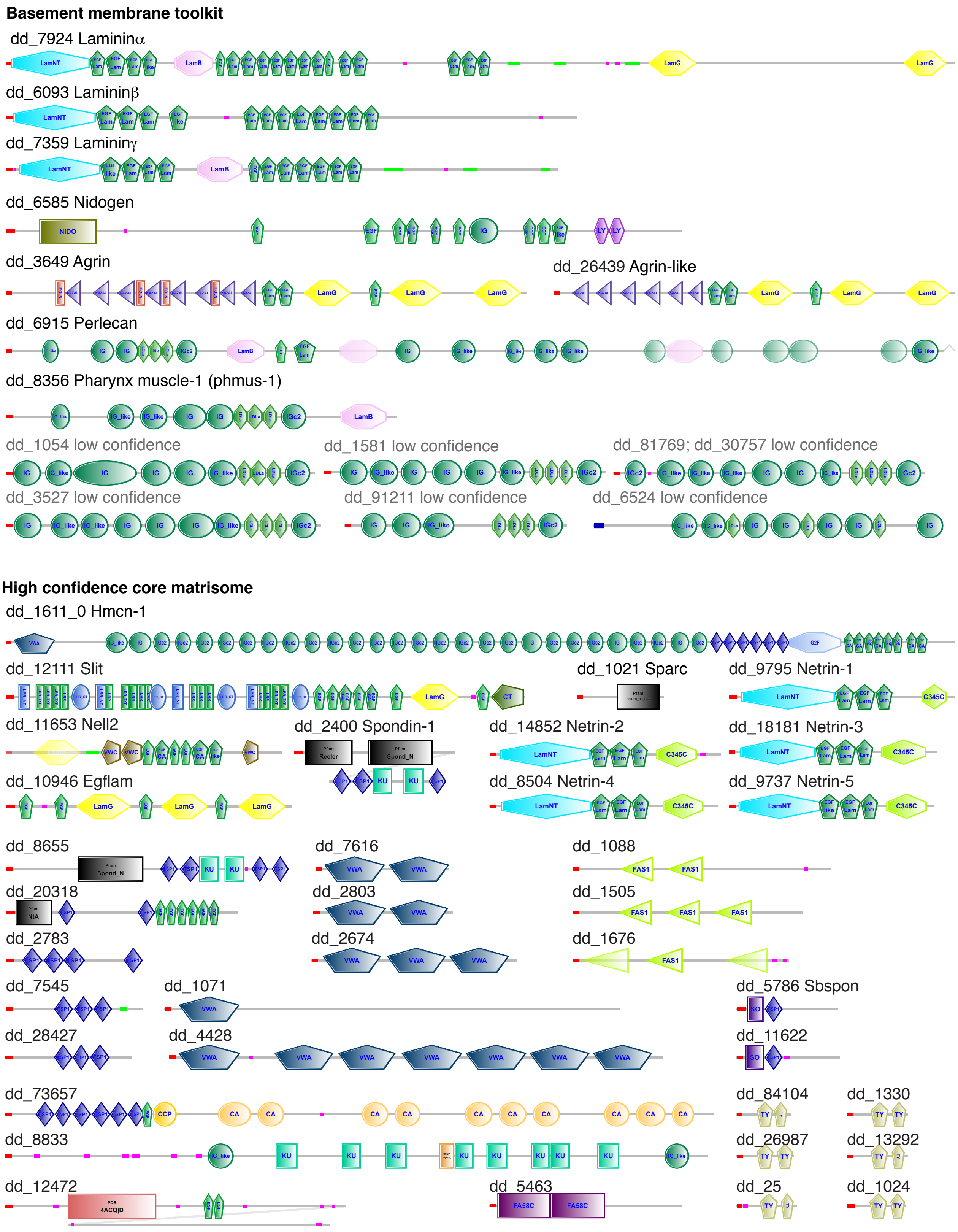

Supplementary Figure 1

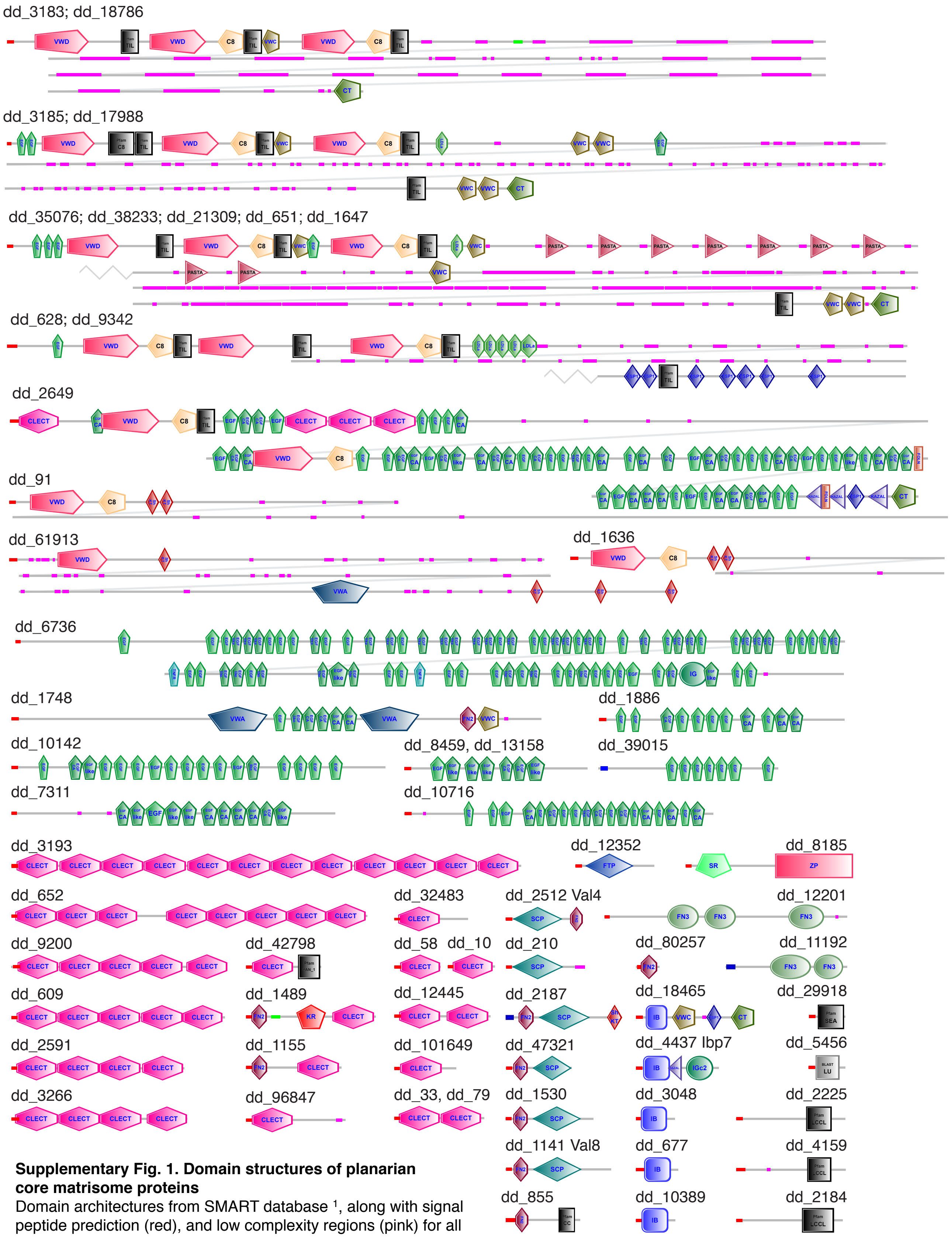

Supplementary Fig. 1. Domain structures of planarian core matrisome proteins
Domain architectures from SMART database [1], along with signal peptide prediction (red), and low complexity regions (pink) for all predicted planarian core glycoproteins. Grey straight lines indicate continuation of coding sequence to the next line while grey peaked lines indicate that the protein sequences shown are likely part of the same coding sequence however the potential splicing junctions in genomic sequence are unclear.

Supplementary Figure 2

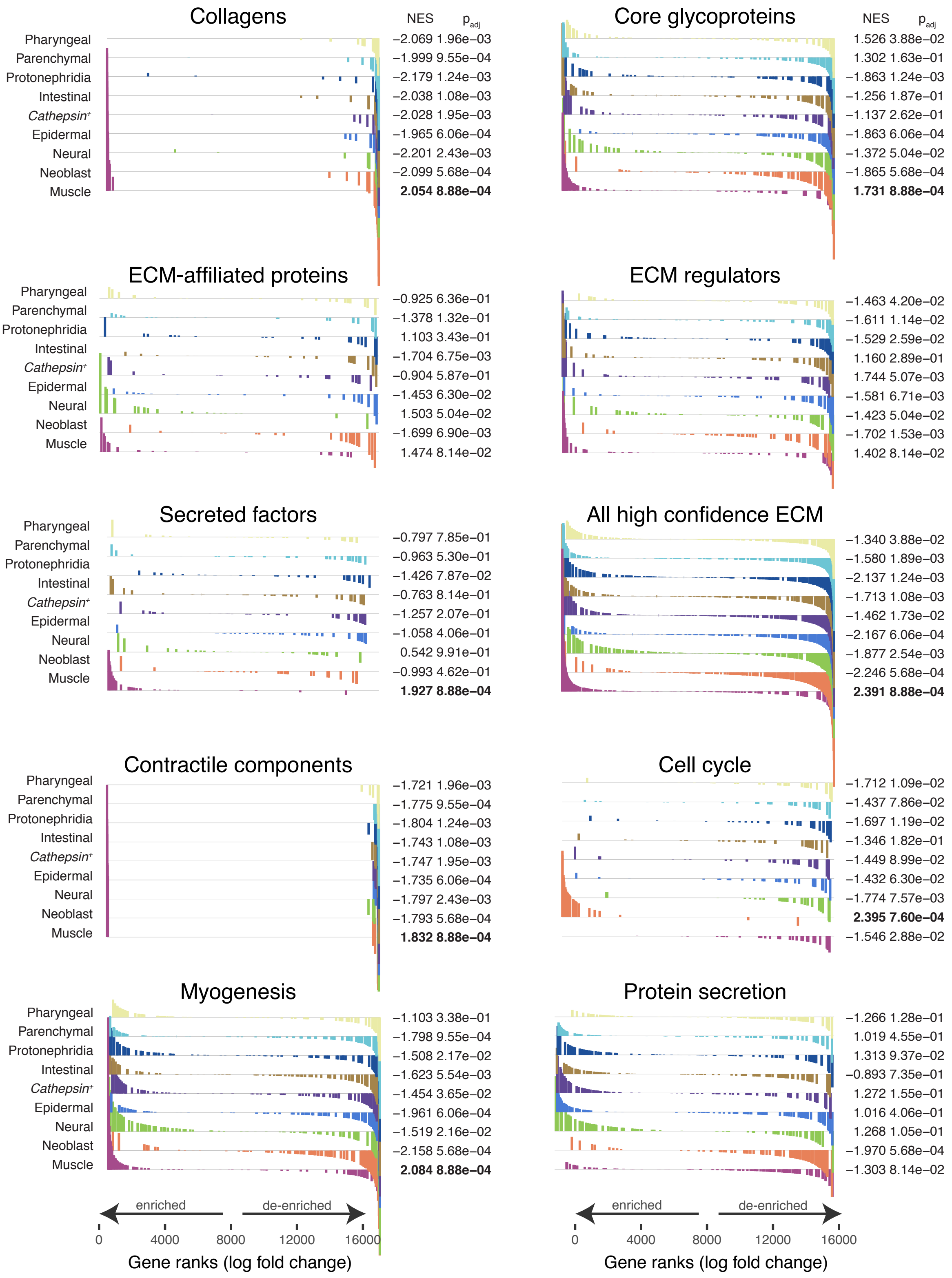

Supplementary Fig. 2. Gene set enrichment analysis shows enrichment of matrisome genes in muscle cells
Graphs show gene set enrichment for indicated matrisome or control gene sets (Supplementary Table 3) in different cell types. Average log fold change of each cell type vs all other cells was ranked, and the genes within each gene set are indicated by bars and colored by cell type as in Fig. 2a. Normalized enrichment score and adjusted p-values are indicated beside each graph with significant enrichment indicated in bold.

Supplementary Figure 3

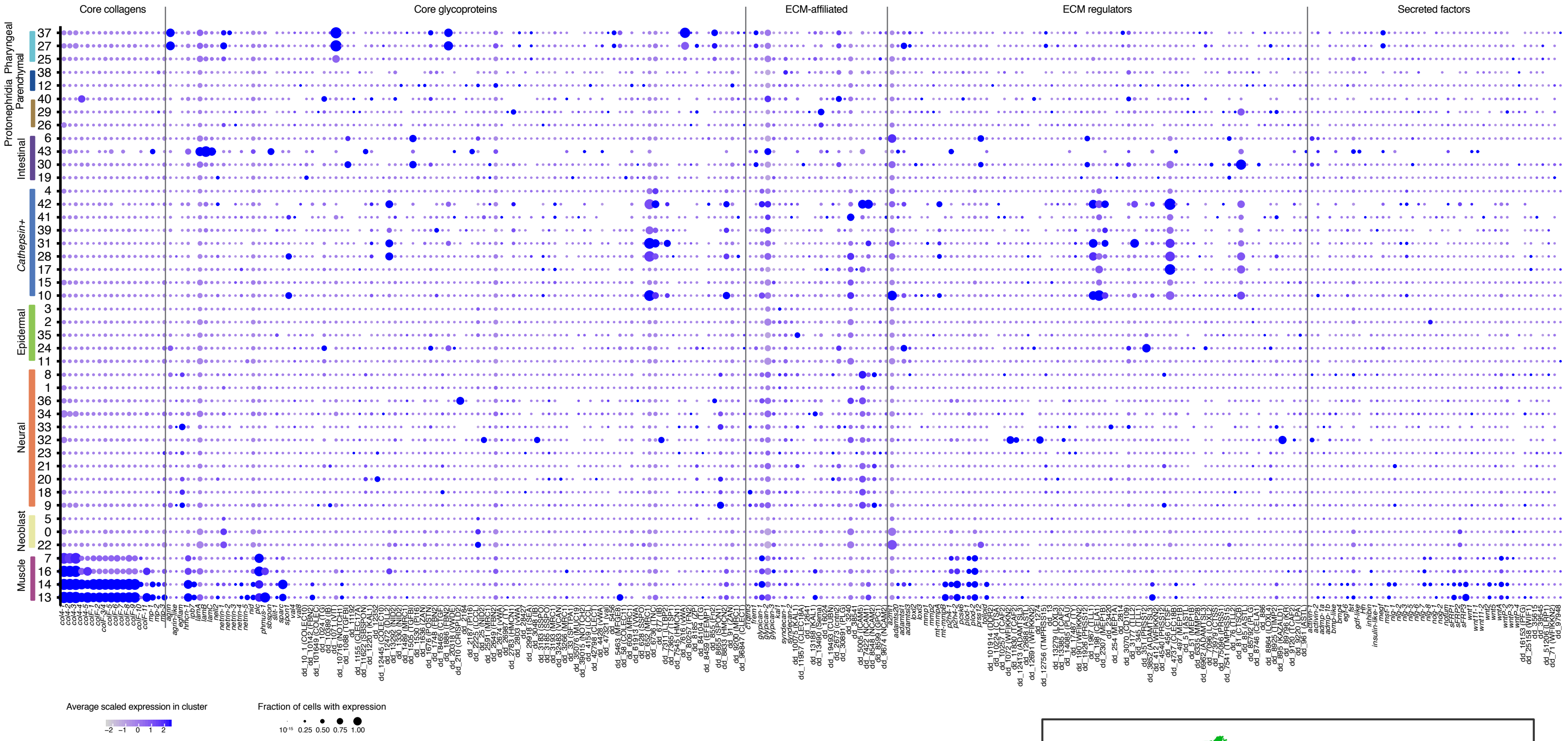

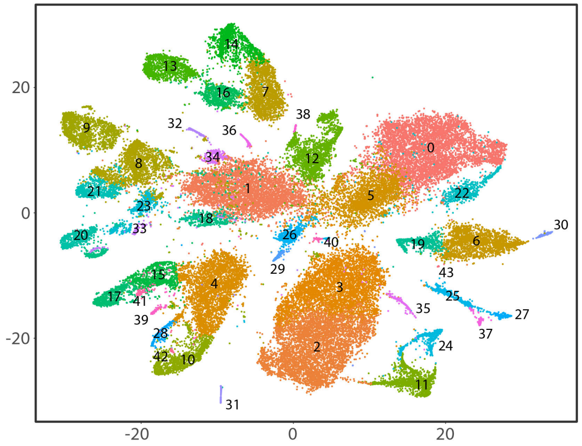

Supplementary Fig. 3. Expression of matrisome components in different cell types using SCS data

Each circle within the table represents the expression of a gene (column) in a particular cell type (row) within the SCS data. Each circle is colored by average expression across expressing cells within the cell type and sized proportional to the percentage of cells within the cell type that express the gene. The expression of high confidence matrisome genes in the 44 clusters of the entire SCS data, numbered as shown in the tSNE plot [2].

Supplementary Figure 4

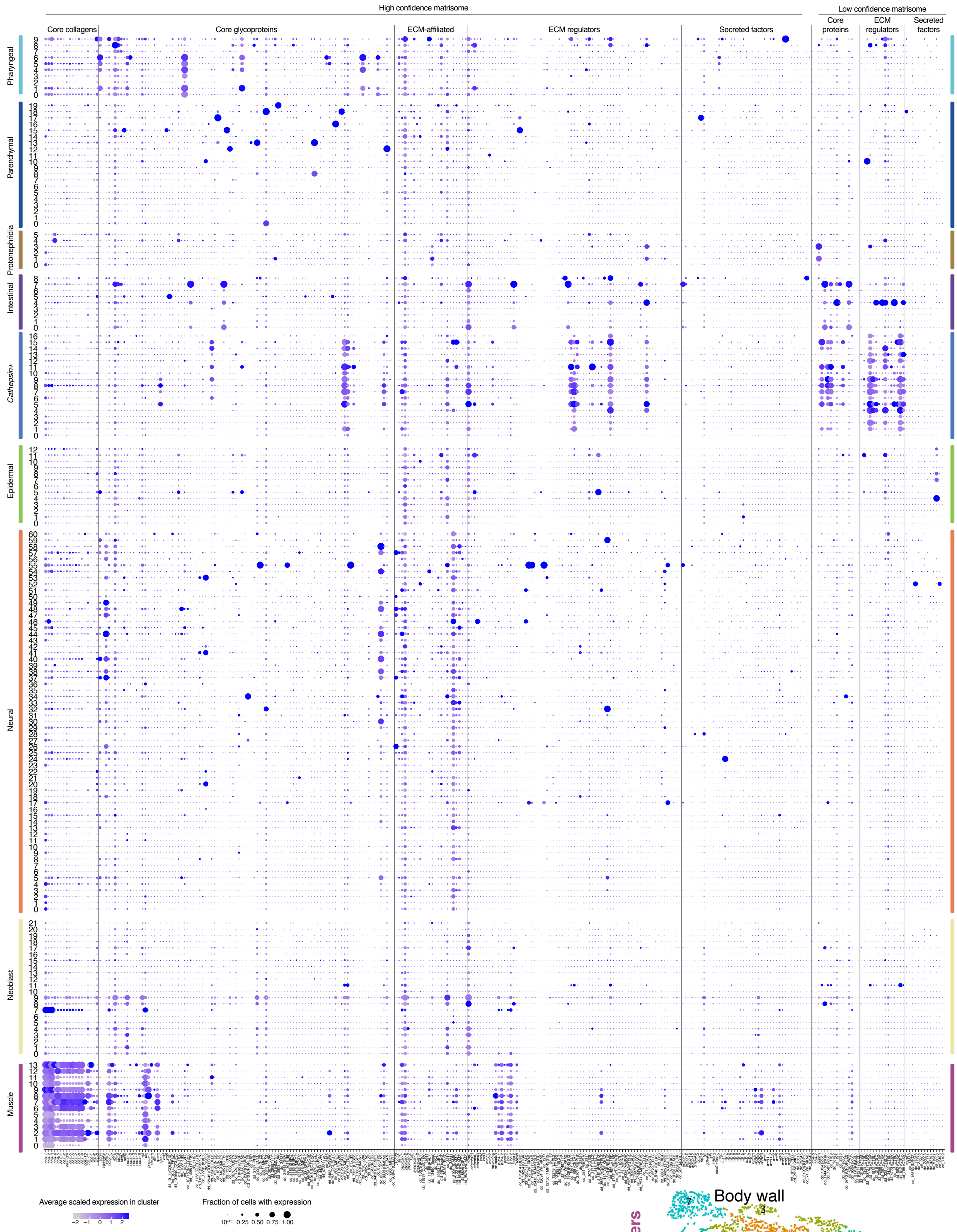

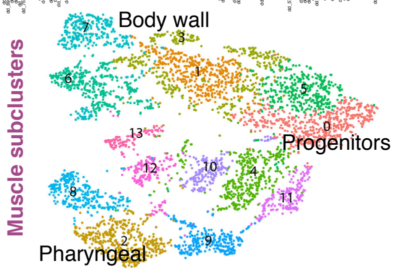

Supplementary Fig. 4. Expression of matrisome components in different cell subtypes using SCS data

Each circle represents the expression of a gene (column) in a particular cell subtype (row) within the SCS data, colored by average expression across expressing cells within the cell subtype and sized proportional to the percentage of cells within the cell subtype that express the gene. Expression of all matrisome genes in the 142 cell-type subclusters, including within the muscle subcluster tSNE plot shown [2].

Supplementary Figure 5

a

column+row+/ row+ total	*colF-1*	*colF-2*	*colF-3*	*colF-4*	*colF-5*	*colF-6*	*colF-7*	*colF-8*	*colF-9*	*colF-10*	*colF-11*	*col4-1*	*col4-2*	*col4-3*	*col4-4*	*col4-5*	*mp-1*	*mp-2*	*mp-3*	*mhc-1*
colF-1		112/112 100%	n.d.	n.d.	n.d.	n.d.	n.d.	n.d.	n.d.	n.d.	n.d.	n.d.	n.d.	n.d.	n.d.	n.d.	n.d.	n.d.	n.d.	n.d.
colF-2	112/112 100%		411/413 99.5%	110/110 100%	67/67 100%	487/491 99.2%	263/264 99.6%	264/264 100%	112/112 100%	330/346 95.4%	124/126 98.4%	270/270 100%	112/112 100%	266/270 98.5%	381/386 98.7%	258/275 93.8%	78/80 97.5%	63/67 94.0%	425/480 88.5%	n.d.
colF-3	n.d.	411/465 88.4%		n.d.	n.d.	n.d.	n.d.	n.d.	n.d.	n.d.	n.d.	n.d.	n.d.	n.d.	n.d.	n.d.	n.d.	n.d.	399/465 85.8%	n.d.
colF-4	n.d.	110/110 100%	n.d.		n.d.	n.d.	n.d.	n.d.	n.d.	n.d.	n.d.	n.d.	n.d.	n.d.	n.d.	n.d.	n.d.	n.d.	n.d.	n.d.
colF-5	n.d.	67/68 98.5%	n.d.	n.d.		n.d.	n.d.	n.d.	n.d.	n.d.	n.d.	n.d.	n.d.	n.d.	n.d.	n.d.	n.d.	63/68 92.6%	n.d.	n.d.
colF-6	n.d.	487/497 98.0%	n.d.	n.d.	n.d.		n.d.	n.d.	n.d.	n.d.	n.d.	n.d.	n.d.	n.d.	382/395 96.7%	n.d.	n.d.	n.d.	46/102 45.0%	n.d.
colF-7	n.d.	263/283 92.9%	n.d.	n.d.	n.d.	n.d.		263/283 92.9%	n.d.	n.d.	n.d.	n.d.	n.d.	n.d.	n.d.	n.d.	n.d.	n.d.	n.d.	n.d.
colF-8	n.d.	264/264 100%	n.d.	n.d.	n.d.	n.d.	263/265 99.3%		n.d.	n.d.	n.d.	n.d.	n.d.	n.d.	n.d.	n.d.	n.d.	n.d.	n.d.	n.d.
colF-9	n.d.	112/112 100%	n.d.	n.d.	n.d.	n.d.	n.d.	n.d.		n.d.	n.d.	n.d.	n.d.	n.d.	n.d.	n.d.	n.d.	n.d.	n.d.	n.d.
colF-10	n.d.	330/346 95.4%	n.d.	n.d.	n.d.	n.d.	n.d.	n.d.	n.d.		123/127 96.8%	n.d.	n.d.	n.d.	n.d.	n.d.	n.d.	n.d.	n.d.	n.d.
colF-11	n.d.	124/134 92.5%	n.d.	n.d.	n.d.	n.d.	n.d.	n.d.	n.d.	123/134 91.8%		n.d.	n.d.	n.d.	n.d.	n.d.	n.d.	n.d.	n.d.	n.d.
col4-1	n.d.	270/390 69.2%	n.d.	n.d.	n.d.	n.d.	n.d.	n.d.	n.d.	n.d.	n.d.		n.d.	385/390 98.7%	n.d.	n.d.	n.d.	n.d.	n.d.	131/271 48.34%
col4-2	112/142 78.9%	112/142 78.9%	n.d.	n.d.	n.d.	n.d.	n.d.	n.d.	n.d.	n.d.	n.d.	n.d.		n.d.	n.d.	n.d.	n.d.	n.d.	n.d.	n.d.
col4-3	n.d.	266/385 69.1%	n.d.	n.d.	n.d.	n.d.	n.d.	n.d.	n.d.	n.d.	n.d.	385/385 100%	n.d.		n.d.	n.d.	n.d.	n.d.	n.d.	n.d.
col4-4	n.d.	381/389 97.9%	n.d.	n.d.	n.d.	382/385 99.2%	n.d.	n.d.	n.d.	n.d.	n.d.	n.d.	n.d.	n.d.		n.d.	n.d.	n.d.	n.d.	n.d.
col4-5	n.d.	258/265 97.4%	n.d.	n.d.	n.d.	n.d.	n.d.	n.d.	n.d.	n.d.	n.d.	n.d.	n.d.	n.d.	n.d.		n.d.	n.d.	n.d.	n.d.
mp-1	n.d.	78/78 100%	n.d.	n.d.	n.d.	n.d.	n.d.	n.d.	n.d.	n.d.	n.d.	n.d.	n.d.	n.d.	n.d.	n.d.		n.d.	n.d.	n.d.
mp-2	n.d.	63/67 94.0%	n.d.	n.d.	63/67 94.0%	n.d.	n.d.	n.d.	n.d.	n.d.	n.d.	n.d.	n.d.	n.d.	n.d.	n.d.	n.d.		n.d.	n.d.
mp-3	n.d.	425/510 83.3%	399/442 90.3%	n.d.	n.d.	46/68 67.6%	n.d.	n.d.	n.d.	n.d.	n.d.	n.d.	n.d.	n.d.	n.d.	n.d.	n.d.	n.d.		n.d.
mhc-1	n.d.	n.d.	n.d.	n.d.	n.d.	n.d.	n.d.	n.d.	n.d.	n.d.	n.d.	131/133 98.5%	n.d.	n.d.	n.d.	n.d.	n.d.	n.d.	n.d.	
dd_id	dd_1070	dd_702	dd_649	dd_649r	dd_740	dd_832	dd_840	dd_402	dd_701	dd_9565	dd_8075	dd_1579	dd_2197	dd_2337	dd_2500	dd_7811	dd_6811	dd_9030	dd_3516	dd_579

b

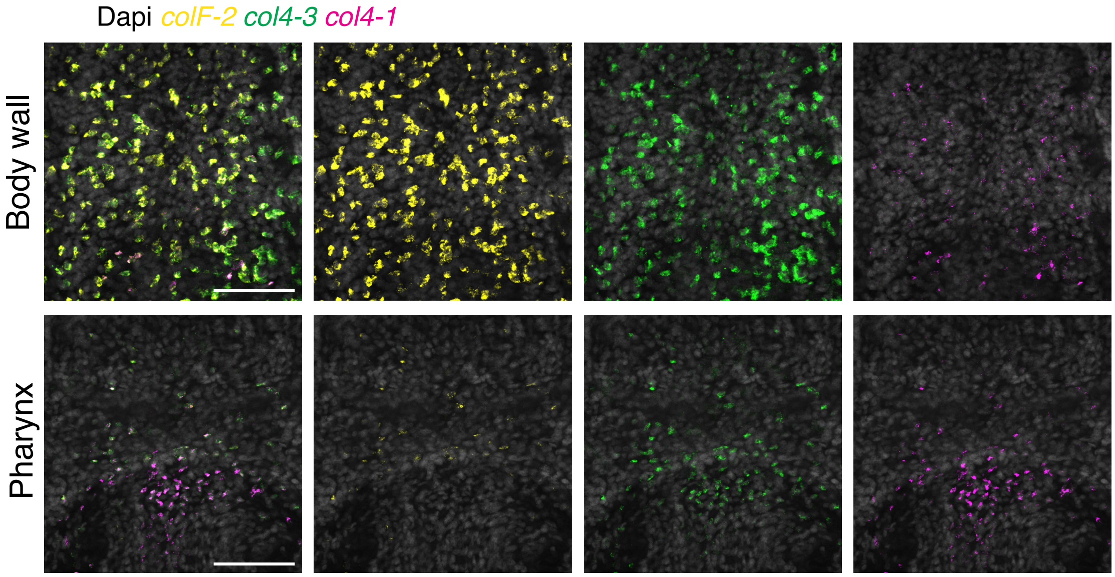

Supplementary Fig. 5. Planarian collagen co-expression demonstrated by FISH

a Table shows co-expression numbers (double positive/row single positive) and percentages. Counts reflect co-expression numbers counted on the ventral side in 1 animal as representative of 4 animals. Shade of blue is proportional to co-expression percentage. **b** Single confocal slices of collagen type IV expression in the body-wall with *colF-2* [3] and expression in the pharynx. This is likely pharynx muscle given co-expression with *mhc-1* as shown in **a**. Images representative of 4 animals. Scale bar, 100 µm.

Supplementary Figure 6

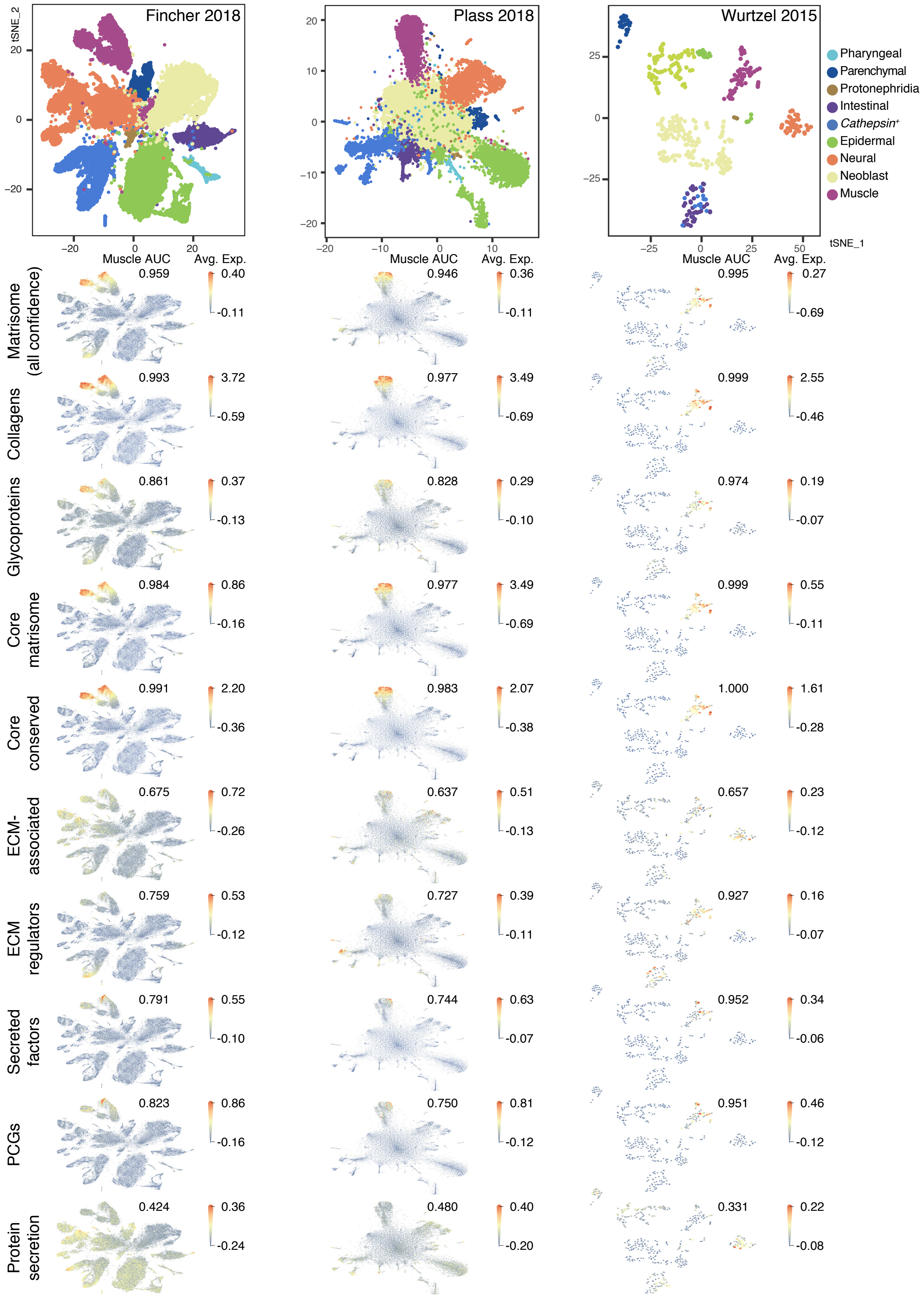

Supplementary Fig. 6. Muscle is the major source of ECM in independent datasets
The muscle cluster is defined by expression of genes encoding different matrisome subsets, using high confidence matrisome genes except for the first row, secreted PCGs, and genes encoding homologs of protein secretion machinery as shown by average expression of the gene set within each cell. Area under curve (AUC) value from roc analysis (false positive rate vs true positive rate) shows the ability of the average expression module shown to correctly classify muscle cells. For completeness, all plots are shown here, duplicating images shown in Fig. 2a,d and Figure 6. Data from Fincher et al, 2018 [2], Plass et al, 2018 [4], and Wurtzel et al, 2015 [5].

Supplementary Figure 7

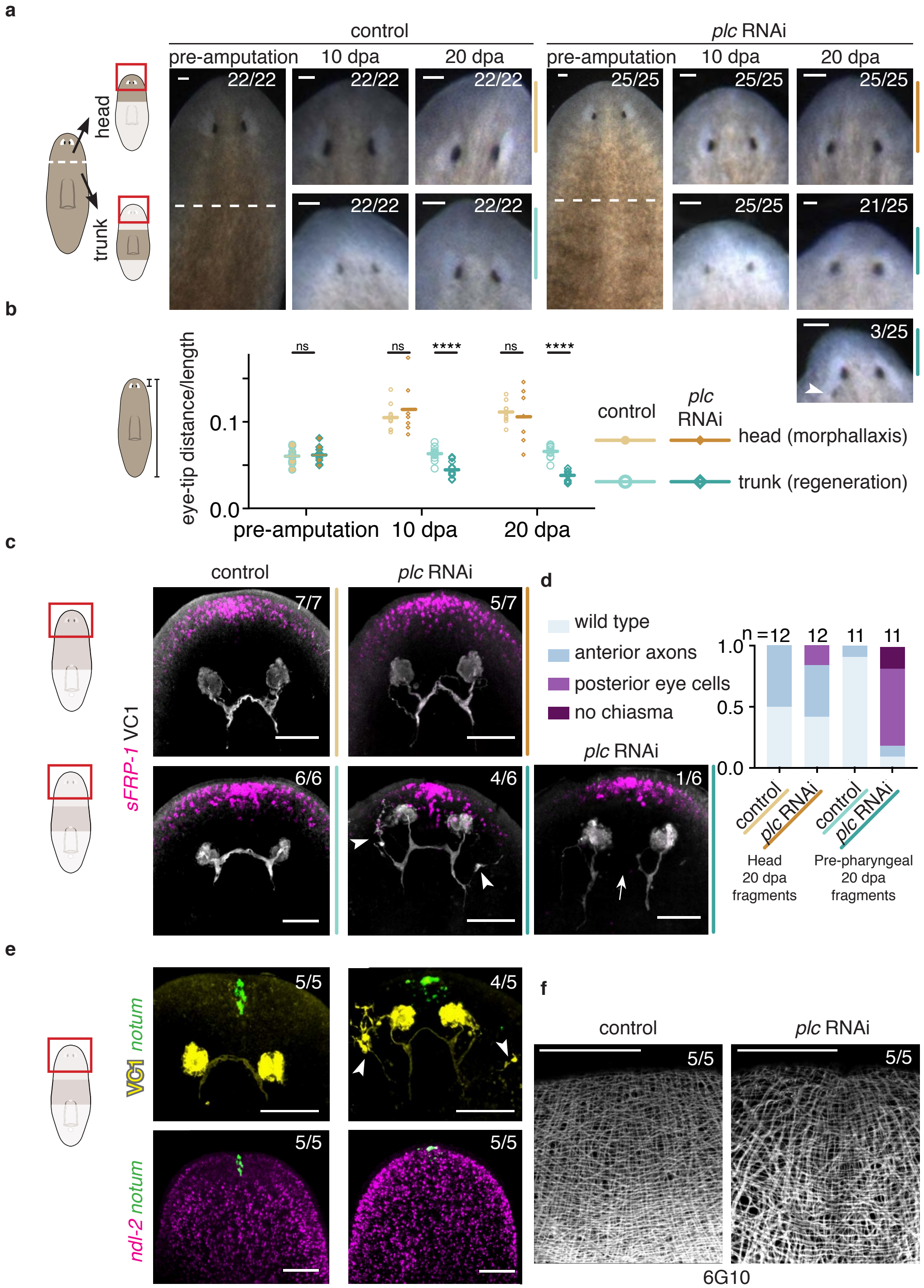

Supplementary Figure 7. Inhibition of *perlecan* leads to defects in head tip regeneration
a Live images of heads during regeneration. A single animal, shown pre-amputation, was amputated (white dashed line) into head and trunk pieces that were then imaged over time. 3 RNAi experiments **b** Quantification of eye-to-head tip distance (as shown in cartoon and colored lines to the right of images in **a**) normalized by total animal length revealed no difference during tissue morphallaxis of head pieces but significant differences (2 way ANOVA with Sidak's mutliple comparisons test) during tissue regeneration of trunk pieces. Line graph shows mean at each time point. Live image to the far right shows most extreme phenotype of ectopic posterior eyes. **c** Anterior marker (*sFRP-1*) and eye organization (VC1) of head and pre-pharyngeal pieces indicates defects in eye organization during head regeneration after *perlecan (plc)* inhibition. **d** Quantification of eye defects in *plc(RNAi)* regenerants. White arrow indicates lack of optic chiasma. **e** The space between the *notum*+ anterior pole and the eyes or the *ndl-2*+ region is absent in *plc(RNAi).* **f** Head tip muscle fibers (6G10+) are slightly disoranized and sparser in *plc(RNAi)* animals. Unless specified, anterior up, red box in cartoon indicates area imaged, white arrowhead indicates ectopic eye, RNA probes, scale bar 100 μm, amputation performed after 35 days of RNAi, animals fixed 20 days post amputation (dpa), fractions indicate number of total animals from 2 independent RNAi experiments with phenotype shown in representative image.

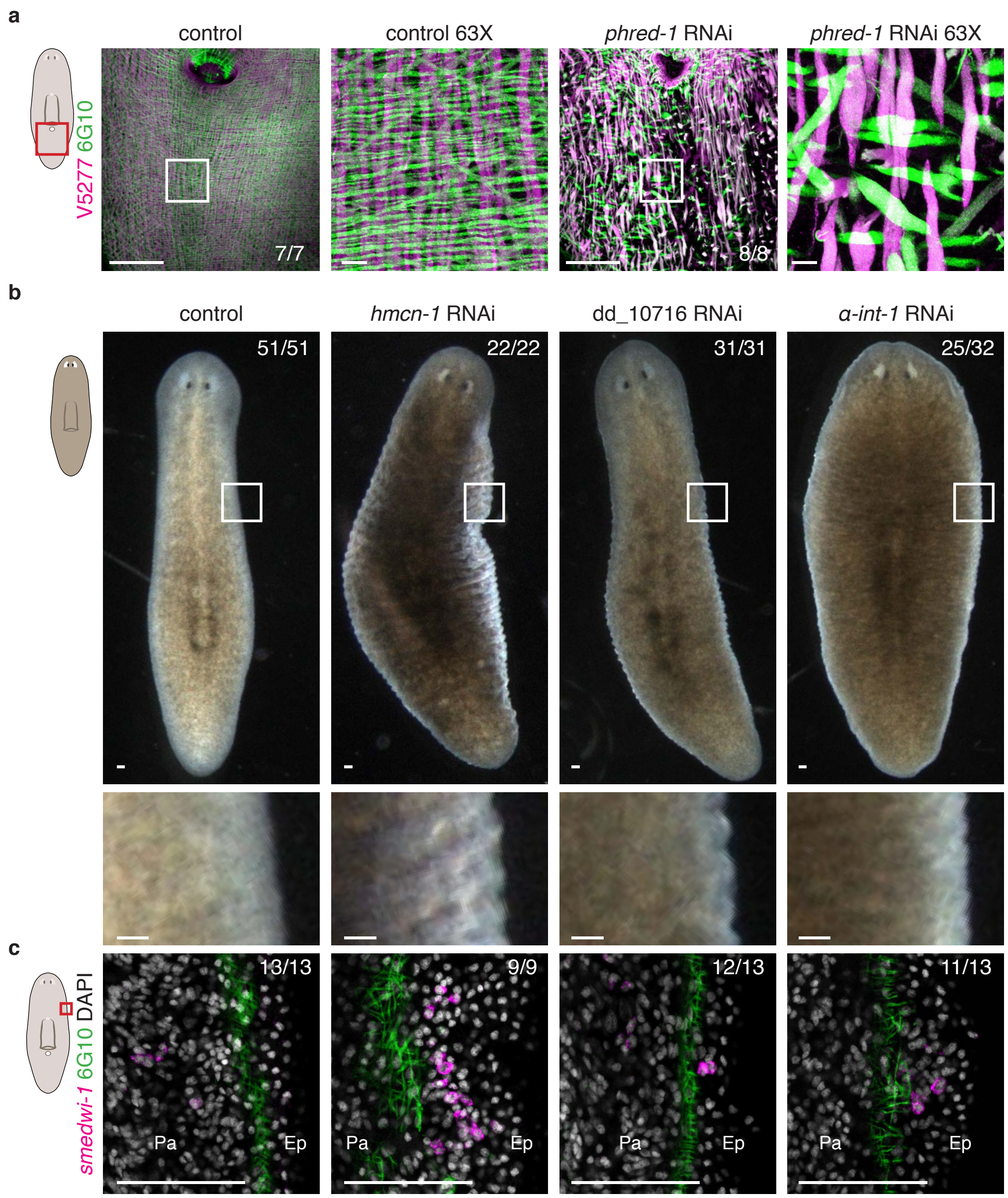

Supplementary Figure 8. Phenotypes of core glycoproteins and receptors predicted to interact with the ECM

a Muscle fibers in the bodywall (circular 6G10⁺ fibers and longitudinal V5277⁺ fibers) are not elongated in *phred-1*(RNAi) animals, similar to previously published data on thickened pharyngeal muscle fibers in in *phred-1*(RNAi) animals [6]. 63X image scale bar, 10 µm. **b** Three RNAi conditions led to varying degrees of epidermal ruffling. dd_10716 encodes an EGF-repeat protein. **c** Ectopic cells, including *smedwi-1*⁺ neoblasts normally confined to the parenchyma (Pa), were present between the 6G10⁺ muscle fiber layer and the outer epidermis (Ep). Unless specified, anterior up, red box in cartoon indicates area imaged, white box indicates area shown at higher magnification, RNA probes, scale bar 100 µm, 33 days of RNAi with 9 feedings during tissue homeosta-sis, fractions indicate number of total animals from 2 independent RNAi experiments with phenotype shown in representative image.

Supplementary Figure 9

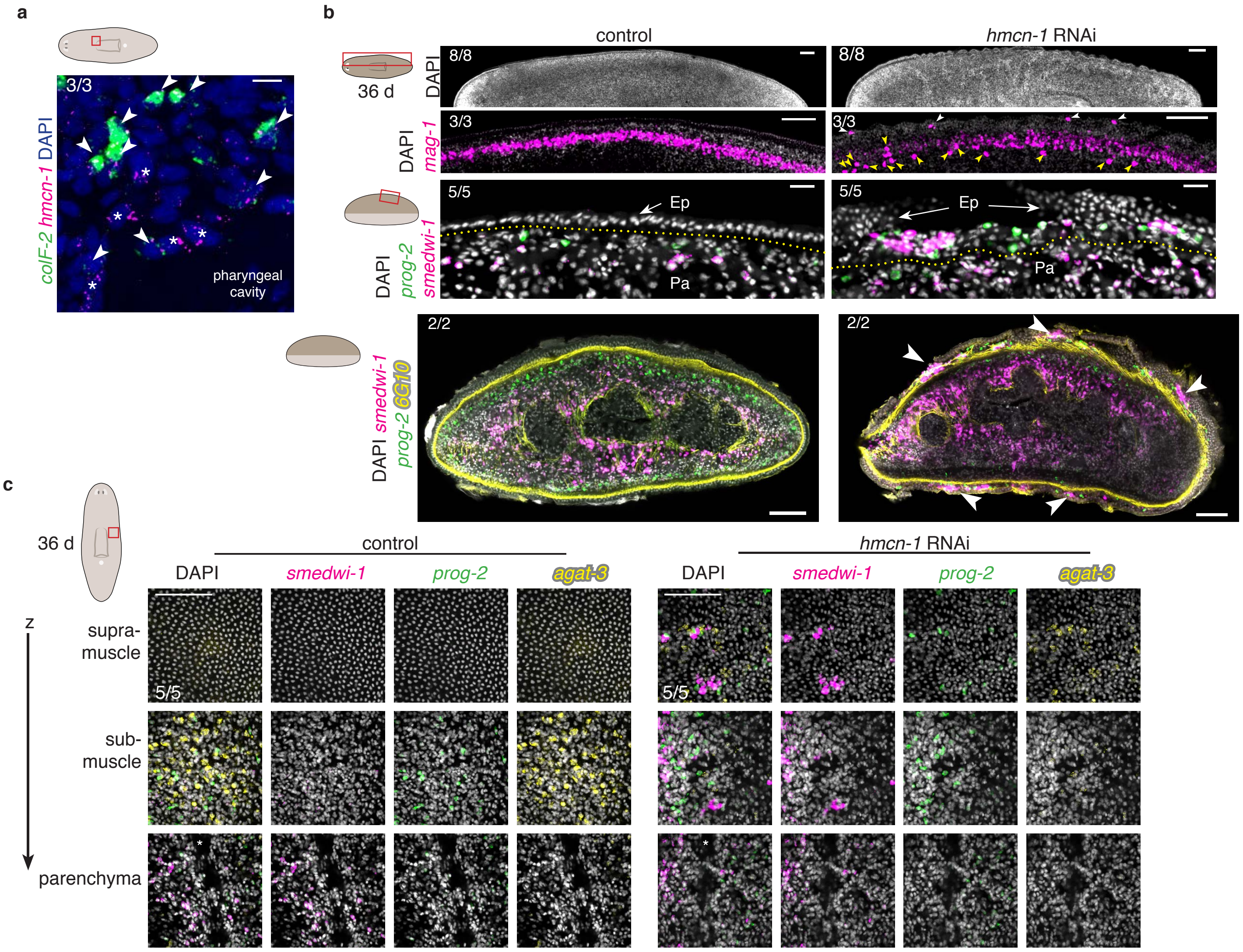

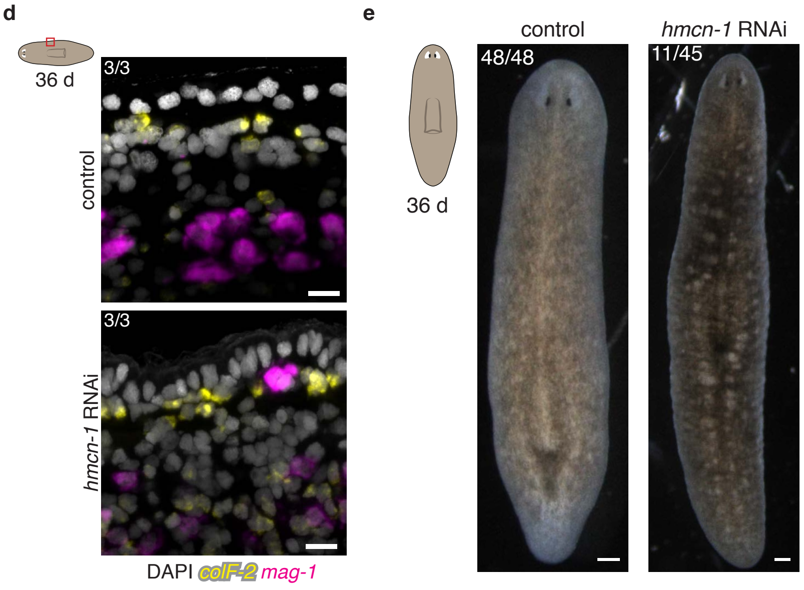

Supplementary Figure 9. Inhibition of *hmcn-1* leads to ectopic neoblasts. a The small number of *colF-2*$^-$ *hmcn-1*$^+$ cells are found near the pharynx cavity. Scale bar 10 μm. **b** Stainings of nuclei (Dapi), neoblasts (*smedwi-1*), epidermal lineage cells (*prog-2*), and secretory cells (*mag-1*) show that inhibition of *hmcn-1* results in ectopic cell localization outside the parenchyma along the anterior-posterior and dorsal-ventral axes. Portions of these images are shown in Fig. 4e. **c** Individual channels of Figure 4d, right panel, showing the expression of neoblasts and neoblast progenitors at various z planes. **d** Ectopic secretory and muscle cells are found above the negative space normally occupied by muscle fibers. Scale bar 10 μm. **e** Live images of worms after 36 days of RNAi. Total number of animals with unpigmented spots from 3 independent RNAi experiments. Unless specified, anterior up, RNA probes, scale bar 100 μm, 36 days RNAi, fractions indicate number of total animals from 2 independent RNAi experiments with phenotype shown in representative image.

Supplementary Figure 10

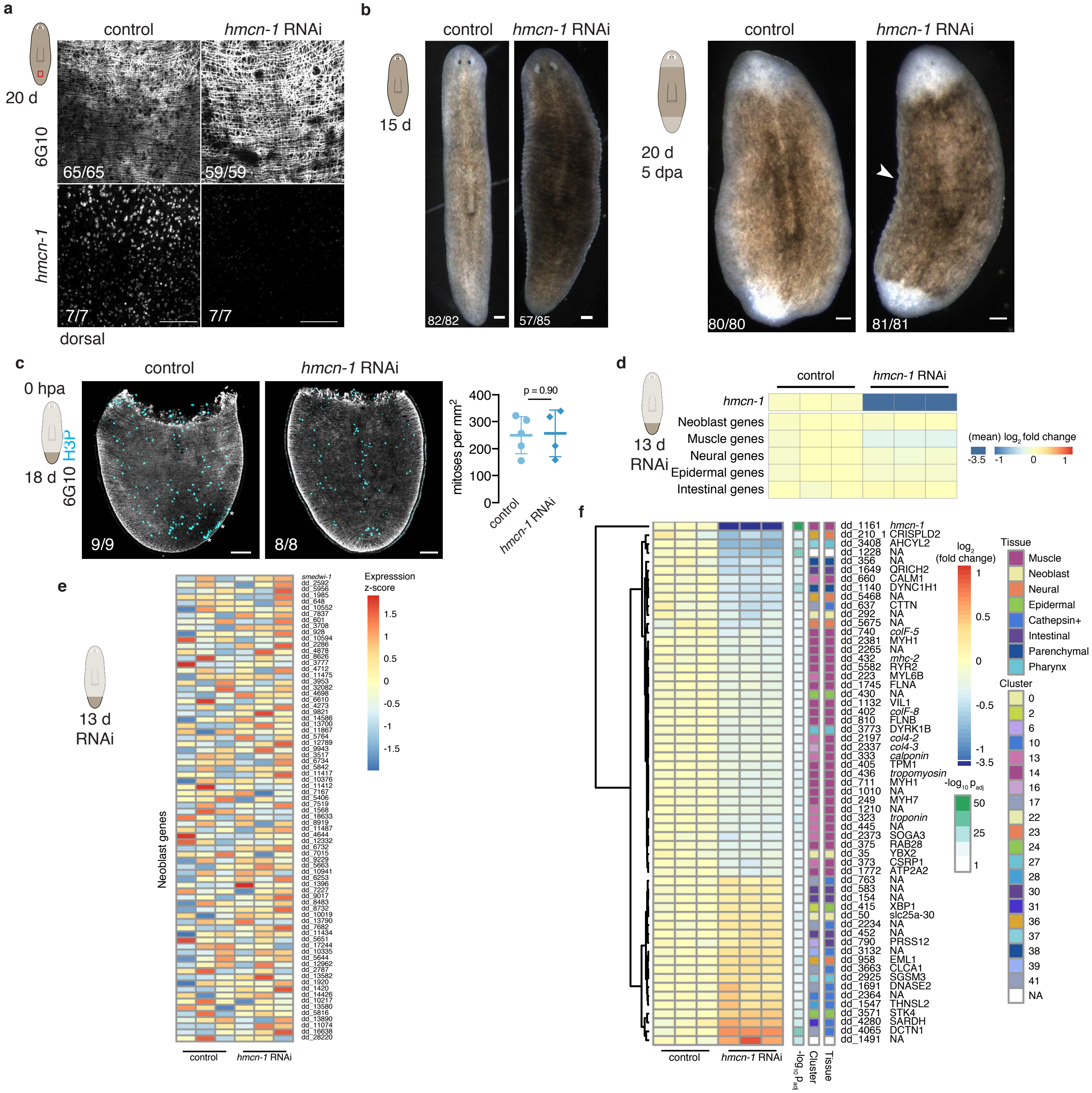

Supplementary Figure 10. Inhibition of *hmcn-1* at early timepoints does not disrupt neoblasts
a *hmcn-1*(RNAi) animals at 20 days of RNAi show a slight disorganization of 6G10⁺ muscle fibers and absence of *hmcn-1*. **b** At this early timepoint, *hmcn-1*(RNAi) animals show slight ruffling (4 RNAi experiments, 1 with 30% penetrance otherwise 100%) and normal blastema formation. Scale bar 200 μm. **c** H3P⁺ (* indicates debris) dividing neoblasts shown as mean with standard deviation (Two-sided Student's t-test). Scale bar 100 μm. **d-f** mRNA-sequencing of three biological replicates (columns) of pooled tail fragments. **d,f** Heatmap of the $\log_2$ fold change of *hmcn-1*(RNAi) over control animals for expression of individual genes (**d**, *hmcn-1*, **f**) or for an average of all genes marking a specific cell type (genes with AUC > 0.8) [3]. **e** Neoblast genes (averaged in **d**), shown as expression z-score show little change in *hmcn-1*(RNAi). **f** All genes identified by DESeq2 with $p_{adj}<0.01$ are shown along with $\log_{10} p_{adj}$ value, tissue and specific cell-type cluster where gene is enriched [1]. Unless specified, anterior up, RNA probes, scale bar 10 μm, fractions indicate number of total animals from 2 independent RNAi experiments with phenotype shown in representative image.

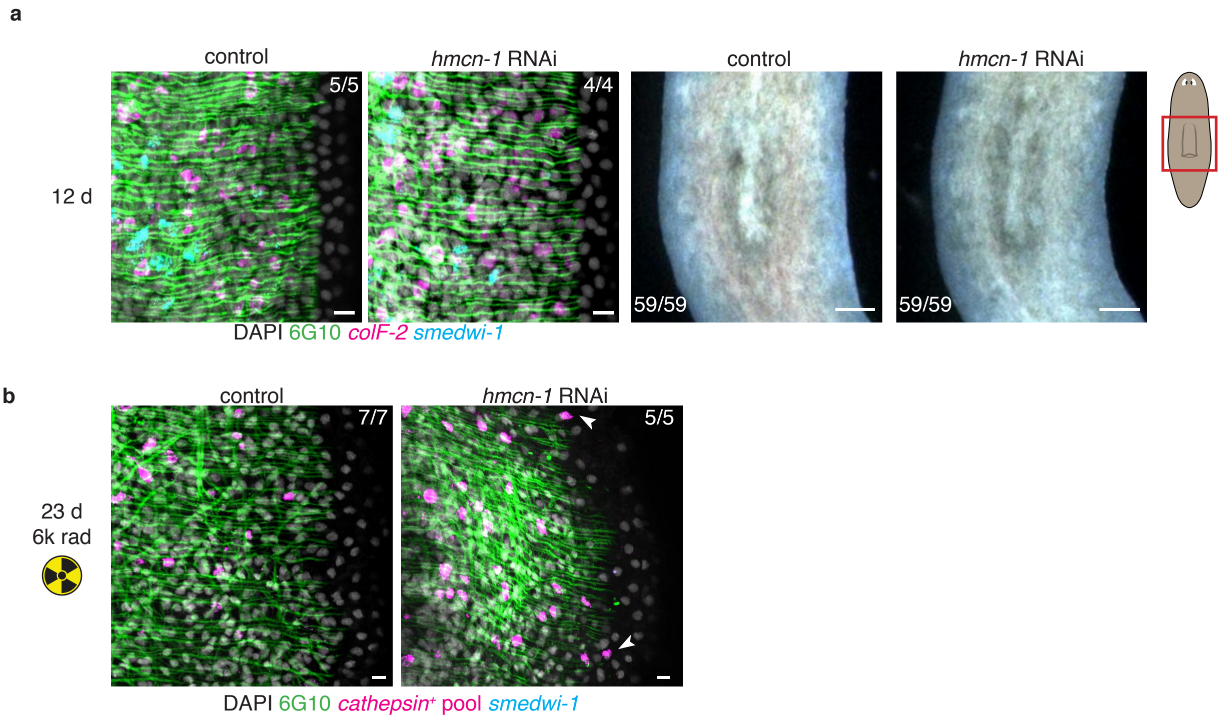

Supplementary Figure 11. Inhibition of *hmcn-1* results in ectopic cell localization independent of neoblasts

a *hmcn-1*(RNAi) animals at 12 days of RNAi do not show ectopic neoblasts or *colF-2*+ muscle nuclei in the supramuscular space or epidermal ruffles in live images. Right scale bar 100 μm. **b** At 23 days of RNAi and 11 days post-irradiation, *hmcn-1*(RNAi) animals also showed ectopic *cathepsin+* cells outside the muscle fiber layer. Unless specified, anterior up, RNA probes, scale bar 10 μm, fractions indicate number of total animals from 2 independent RNAi experiments with phenotype shown in representative image.

Supplementary Figure 12

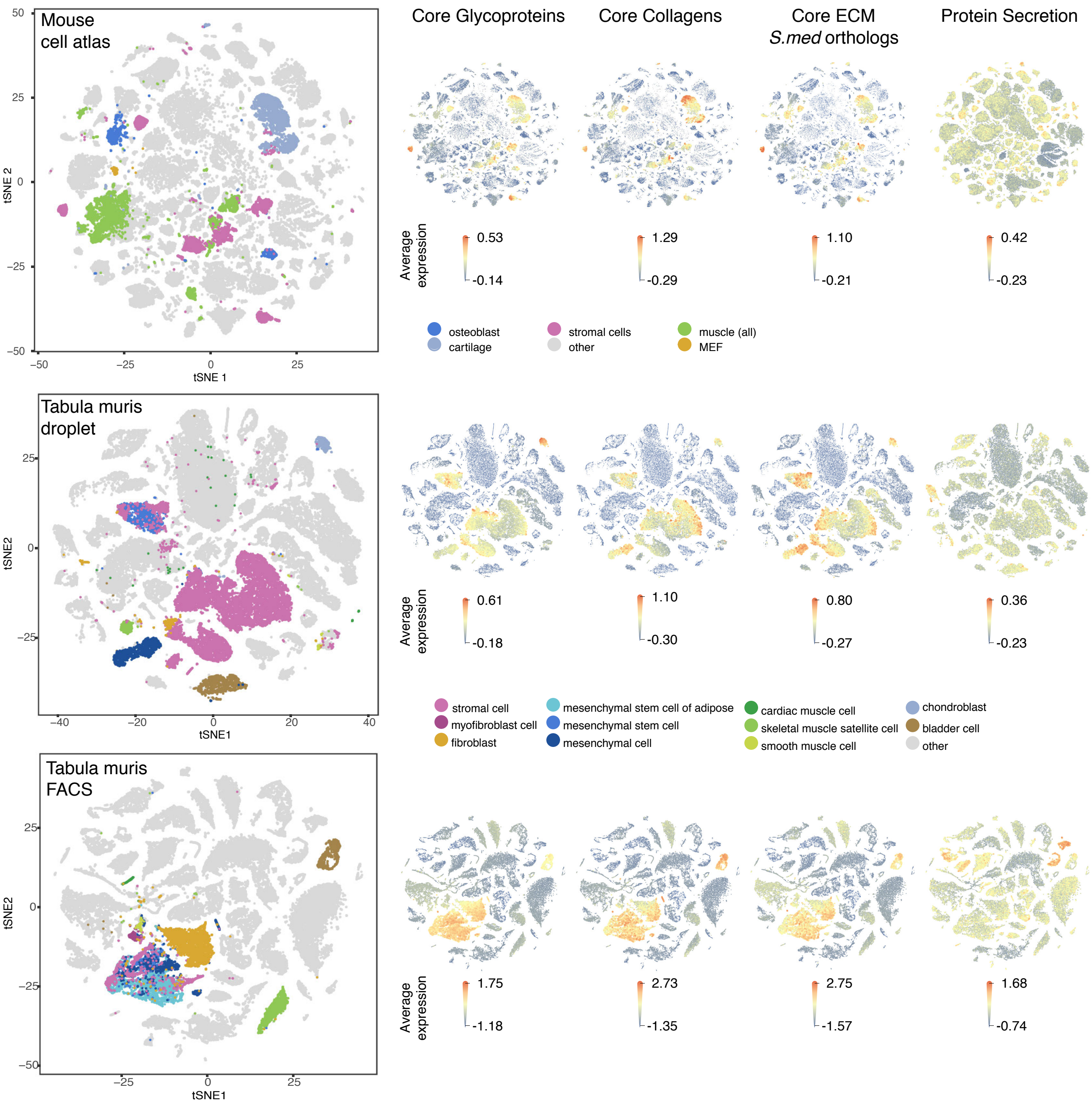

Supplementary Fig. 12. Vertebrate connective tissue expresses ECM components

Murine SCS data from Mouse Cell Atlas [7] and Tabula Muris [8] embedded as tSNE plots was overlaid with average expression of murine matrisome components, including core matrisome components conserved between mouse and planarians, and average expression of genes in the protein secretion module (Supplementary Data 3). Muscle and connective tissues are highlighted in the cell type plots.

Supplementary Figure 13

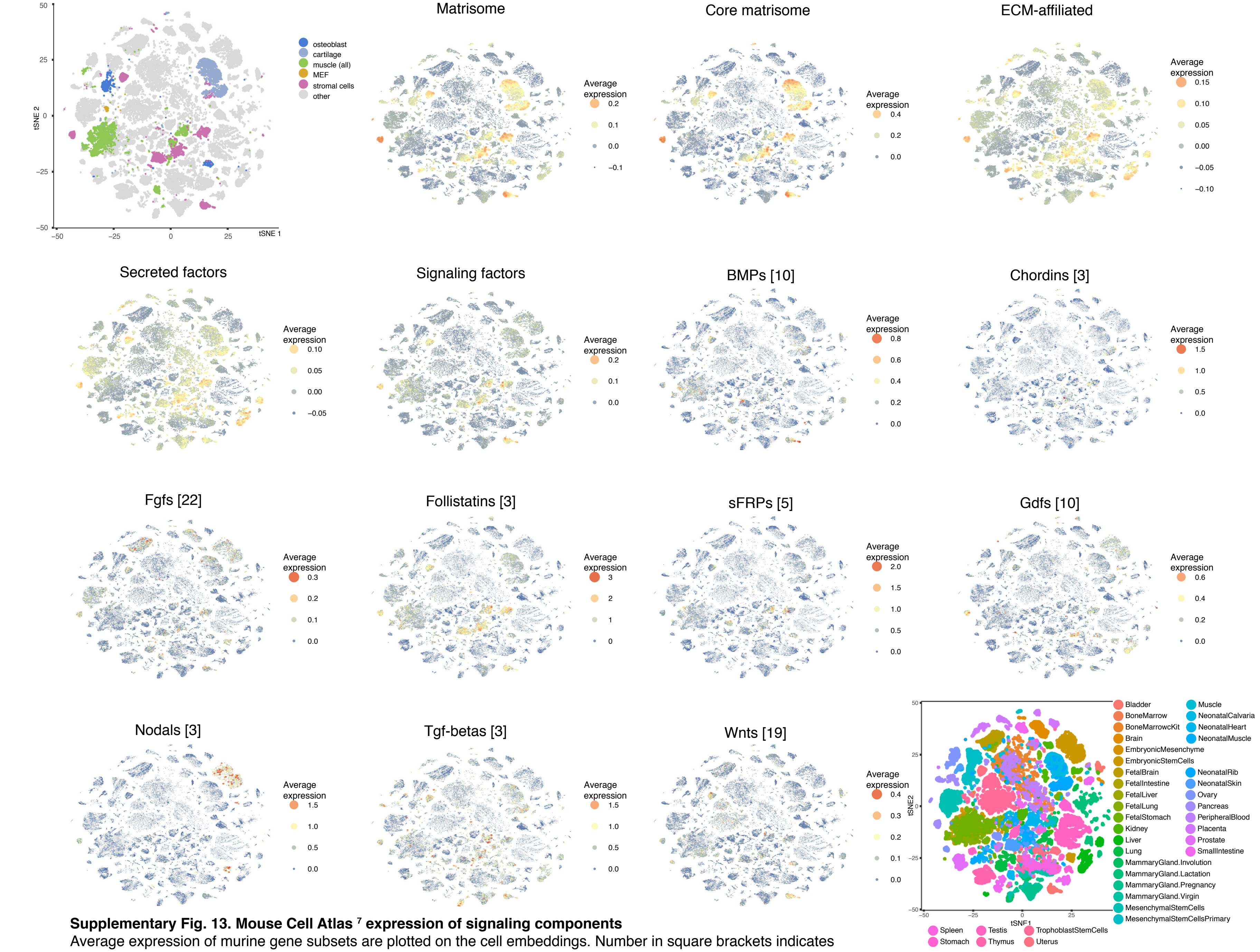

Supplementary Fig. 13. Mouse Cell Atlas [7] expression of signaling components
Average expression of murine gene subsets are plotted on the cell embeddings. Number in square brackets indicates number of ligands averaged for each family of signaling molecules.

Supplementary Figure 14

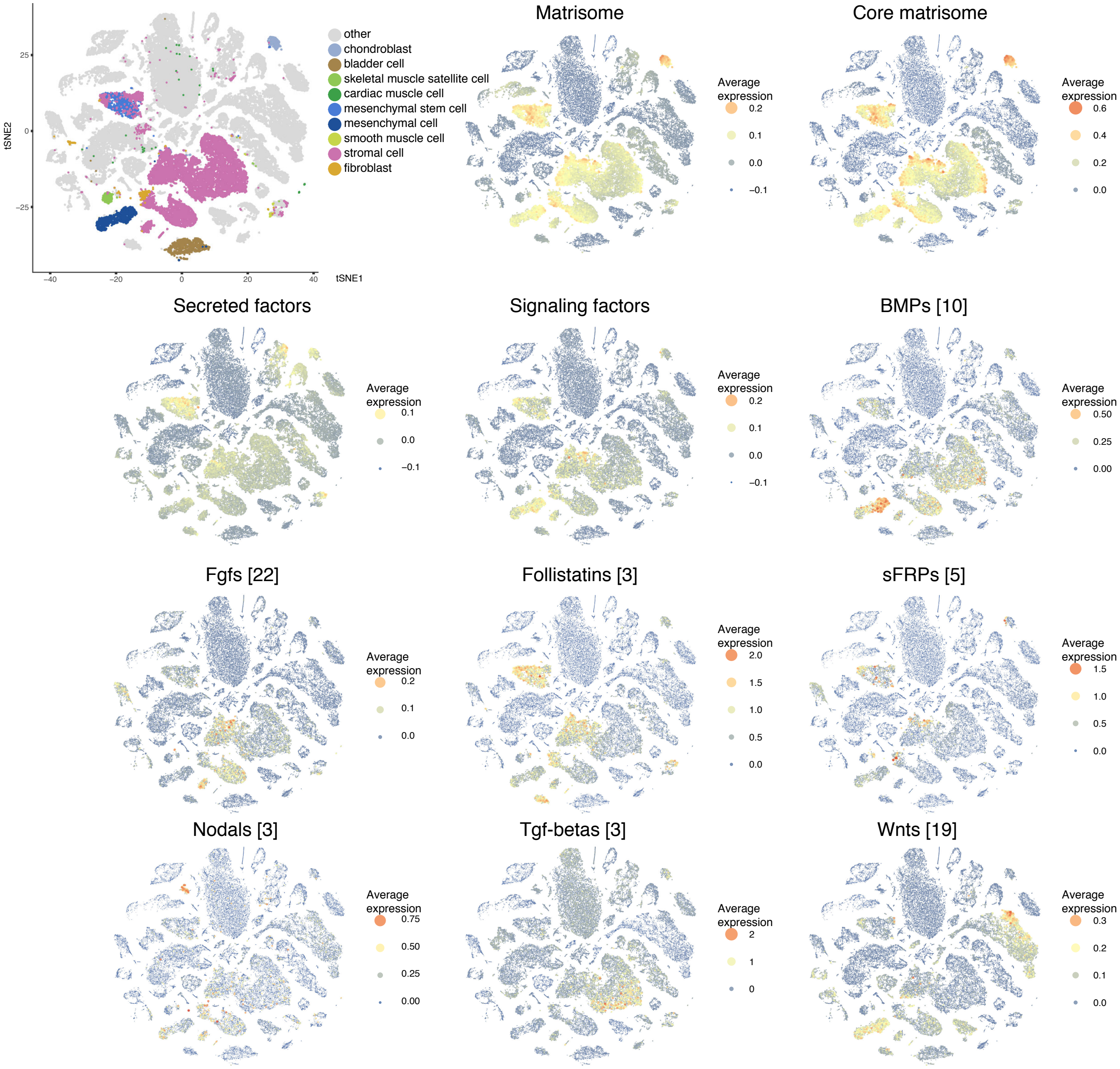

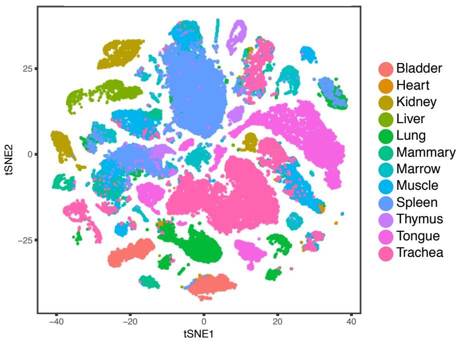

Supplementary Fig. 14. Tabula Muris [8] Droplet SCS data expression of signaling components
Average expression of murine gene subsets are plotted on the cell embeddings. Number in square brackets indicates number of ligands averaged for each family of signaling molecules.

Supplementary Figure 15

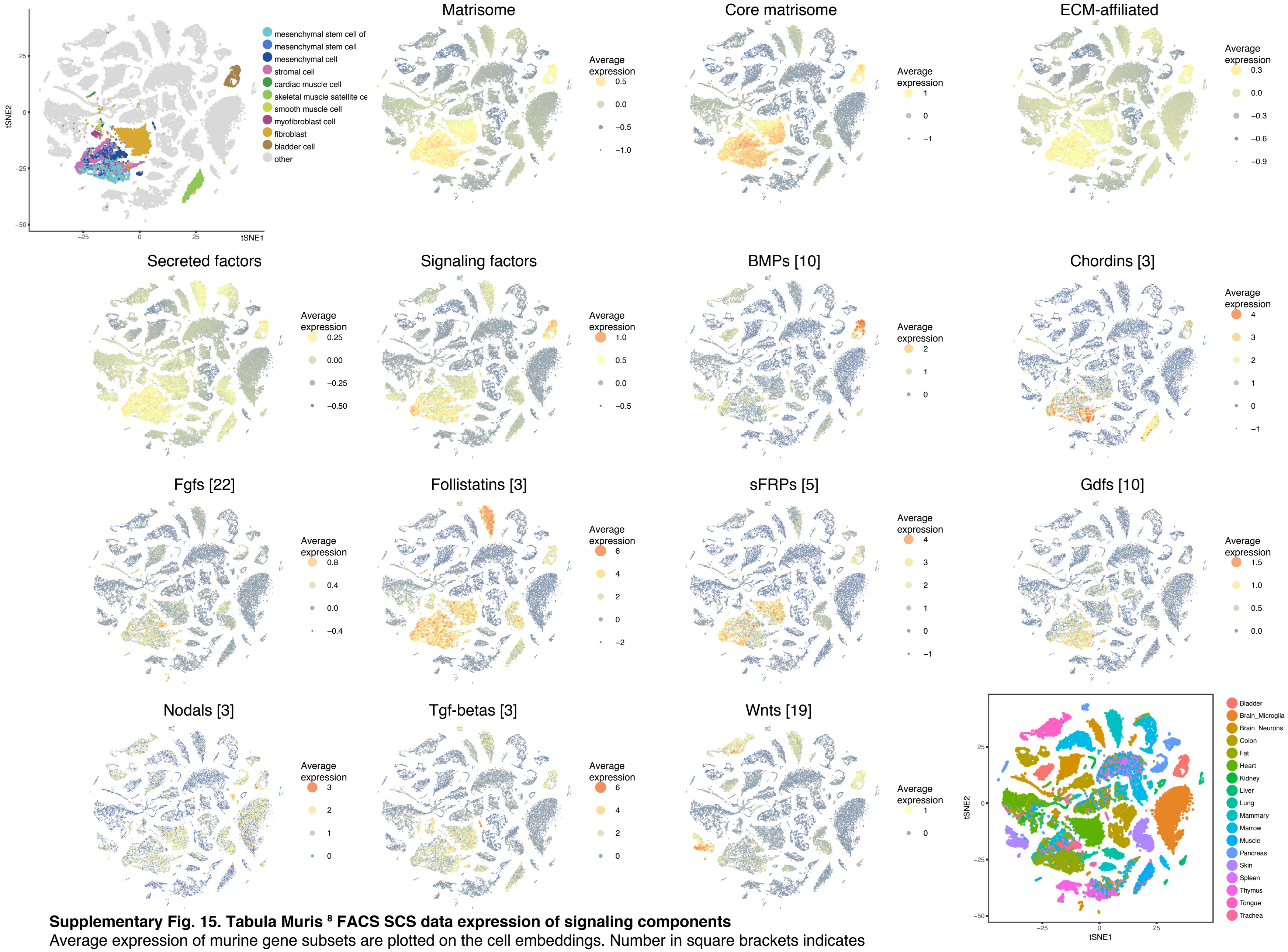

Supplementary Fig. 15. Tabula Muris [8] FACS SCS data expression of signaling components
Average expression of murine gene subsets are plotted on the cell embeddings. Number in square brackets indicates number of ligands averaged for each family of signaling molecules.

Supplementary references

1. Letunic, I., Doerks, T. & Bork, P. SMART: recent updates, new developments and status in 2015. *Nucleic Acids Res* **43**, D257-260 (2015).
2. Fincher, C.T., Wurtzel, O., de Hoog, T., Kravarik, K.M. & Reddien, P.W. Cell type transcriptome atlas for the planarian *Schmidtea mediterranea*. *Science* **360**, eaaq1736 (2018).
3. Witchley, J.N., Mayer, M., Wagner, D.E., Owen, J.H. & Reddien, P.W. Muscle cells provide instructions for planarian regeneration. *Cell Reports* **4**, 633-641 (2013).
4. Plass, M. *et al.* Cell type atlas and lineage tree of a whole complex animal by single-cell transcriptomics. *Science* **360**, eaaq1723 (2018).
5. Wurtzel, O. *et al.* A Generic and Cell-Type-Specific Wound Response Precedes Regeneration in Planarians. *Dev Cell* **35**, 632-645 (2015).
6. Adler, C.E. & Sánchez Alvarado, A. PHRED-1 is a divergent neurexin-1 homolog that organizes muscle fibers and patterns organs during regeneration. *Dev Biol* **427**, 165-175 (2017).
7. Han, X. *et al.* Mapping the Mouse Cell Atlas by Microwell-Seq. *Cell* **172**, 1091-1107 e1017 (2018).
8. Tabula Muris Consortium, T. Single-cell transcriptomics of 20 mouse organs creates a Tabula Muris. *Nature* **562**, 367-372 (2018).
