# Supplementary material for: Muscle functions as a connective tissue and source of extracellular matrix in planarians
Source: Nat Commun. 2019 Apr 8;10:1592. doi: 10.1038/s41467-019-09539-6 (PMC6453901; doi:10.1038/s41467-019-09539-6)
Supplement: Supplementary file 10 — Description of Additional Supplementary Files [file 41467_2019_9539_MOESM10_ESM.pdf]

Title: Supplementary Data 1. Planarian matrisome

Description: Gene names, accession data, matrisome categorization information as described in methods, longest CDS used to determine protein domain organization, SIP (signal peptide) prediction, TM (transmembrane) domain prediction, and GPI (glycosylphosphatidylinositol) anchor predictions, and expression in cell clusters for each gene predicted to be part of the matrisome. If more than one contigs formed the same gene, one representative contig is shown while others are labeled at the end of the table.

Title: Supplementary Data 2. Planarian genes excluded from matrisome

Description: Gene names, accession data, initial matrisome categorization information as described in methods, longest CDS used to determine protein domain organization, SIP (signal peptide) prediction, TM (transmembrane) domain prediction, and GPI (glycosylphosphatidylinositol) anchor predictions, and expression in cell clusters for each gene removed from the final planarian matrisome. Contigs were labeled unclear transcript even if a genomic loci was evident if the contig was part of an intron or was not supported by other transcriptome assemblies.

Title: Supplementary Data 3. Gene lists

Description: Lists with gene names, contigs and/or best human homology used for average expression analyses.

Title: Supplementary Data 4. RNAi screen results

Description: Results from preliminary gene inhibition studies on several matrisome genes or receptors likely to interact with the extracellular matrix expressed in muscle cells.

Title: Supplementary Data 5. Differential expression in *hmcn-1*(RNAi) animals
Description: Differential expression analysis by DESeq2 for contigs with $p_{adj} < 0.05$ changed in *hmcn-1*(RNAi) tails versus control tails after 13 days of RNAi during homeostatic tissue turnover along with cell cluster data.

Title: Supplementary Data 6. Primers and accession numbers for genes cloned
Description: Primers used for nested PCR to clone genes used for *in situ* hybridization or RNAi analysis and accession numbers for Genbank.

Title: Supplementary Movie 1. Neoblasts and muscle in control animals
Description: The expression of the neoblast marker *smedwi-1* (magenta), muscle antibody 6G10 (green), and DAPI (grey) is shown in a 63X confocal z-stack of a control animal at 20 days of RNAi. z-stack starts on the ventral side. A single frame from this movie is shown in Fig. 5b.

Title: Supplementary Movie 2. Neoblasts and muscle in *hmcn-1(RNAi)* animals
Description: The expression of the neoblast marker *smedwi-1* (magenta), muscle antibody 6G10 (green), and DAPI (grey) is shown in a 63X confocal z-stack of a *hmcn-1(RNAi)* animal at 20 days of RNAi. z-stack starts on the ventral side. A single frame from this movie is shown in Fig. 5b.
