# Supplementary material for: Muscle functions as a connective tissue and source of extracellular matrix in planarians
Source: Nat Commun. 2019 Apr 8;10:1592. doi: 10.1038/s41467-019-09539-6 (PMC6453901; doi:10.1038/s41467-019-09539-6)
Supplement: Supplementary file 11 — Reporting Summary [file 41467_2019_9539_MOESM11_ESM.pdf]

natureresearch

Corresponding author(s): Peter W. Reddien

Reporting Summary

Nature Research wishes to improve the reproducibility of the work that we publish. This form provides structure for consistency and transparency in reporting. For further information on Nature Research policies, see Authors & Referees and the Editorial Policy Checklist.

Statistical parameters

When statistical analyses are reported, confirm that the following items are present in the relevant location (e.g. figure legend, table legend, main text, or Methods section).

n/a	Confirmed	
☐	☒	The exact sample size (*n*) for each experimental group/condition, given as a discrete number and unit of measurement
☐	☒	An indication of whether measurements were taken from distinct samples or whether the same sample was measured repeatedly
☐	☒	The statistical test(s) used AND whether they are one- or two-sided<br>*Only common tests should be described solely by name; describe more complex techniques in the Methods section.*
☒	☐	A description of all covariates tested
☐	☒	A description of any assumptions or corrections, such as tests of normality and adjustment for multiple comparisons
☐	☒	A full description of the statistics including central tendency (e.g. means) or other basic estimates (e.g. regression coefficient) AND variation (e.g. standard deviation) or associated estimates of uncertainty (e.g. confidence intervals)
☐	☒	For null hypothesis testing, the test statistic (e.g. *F*, *t*, *r*) with confidence intervals, effect sizes, degrees of freedom and *P* value noted<br>*Give P values as exact values whenever suitable.*
☒	☐	For Bayesian analysis, information on the choice of priors and Markov chain Monte Carlo settings
☒	☐	For hierarchical and complex designs, identification of the appropriate level for tests and full reporting of outcomes
☒	☐	Estimates of effect sizes (e.g. Cohen's *d*, Pearson's *r*), indicating how they were calculated
☐	☒	Clearly defined error bars<br>*State explicitly what error bars represent (e.g. SD, SE, CI)*

Our web collection on statistics for biologists may be useful.

Software and code

Policy information about availability of computer code

Data collection	Zeiss ZEN 2011 and AxioVision v4.7.1 for image data collection. python 3.6.4 with pandas v0.22.0, numpy v1.14.0, and intermine v1.11.0 were used to collect putative matrisome-encoding genes.
Data analysis	interproscan v5.31, signalP v4.0, predGPI, tmhmm v2.0, SMART (http://smart.embl-heidelberg.de) were used to analyze protein sequences. R 3.4.4 with packages Seurat v2.3, AUC v0.3.0, fgsea v1.4.1, and ggridges v0.4.1 were used for single-cell sequencing analysis. ImageJ v2.0.0 1.52g was used for image analysis and post-processing. Prism 7.0 for statistical analysis. bowtie v 1.2.2, samtools v1.9, and DESeq2 v1.18.1 were used to analyze bulk RNA-sequencing data.

For manuscripts utilizing custom algorithms or software that are central to the research but not yet described in published literature, software must be made available to editors/reviewers upon request. We strongly encourage code deposition in a community repository (e.g. GitHub). See the Nature Research guidelines for submitting code & software for further information.

Data

Policy information about availability of data

All manuscripts must include a data availability statement. This statement should provide the following information, where applicable:

- Accession codes, unique identifiers, or web links for publicly available datasets
- A list of figures that have associated raw data
- A description of any restrictions on data availability

Data for gene sequences used are provided as web links in Supplementary Data 1, Supplementary Data 2 to unique identifiers available at http://planmine.mpi-cbg.de.
RNA-sequencing is available at NCBI GEO under GSE119840.
Gene sequences are available at Genbank MK430143 - MK430176.
Previously published single-cell RNA-sequencing data sources are listed in Data Availability.

Field-specific reporting

Please select the best fit for your research. If you are not sure, read the appropriate sections before making your selection.

☒ Life sciences ☐ Behavioural & social sciences ☐ Ecological, evolutionary & environmental sciences

For a reference copy of the document with all sections, see nature.com/authors/policies/ReportingSummary-flat.pdf

Life sciences study design

All studies must disclose on these points even when the disclosure is negative.

Sample size	No sample size calculations were performed. Sample sizes were chosen to be similar to previously published data for highly penetrant phenotypes.
Data exclusions	No data was excluded from analyses, except as described in Methods for Supplementary Figure 7b.
Replication	Single-cell sequencing data analysis was performed on three independently previously-generated datasets and found similar results. All experimental results were confirmed with at least two independent RNAi experiments, as indicated.
Randomization	Animals were randomly assigned to RNAi condition and stainings.
Blinding	Investigators were not blinded during data acquisition or analysis, except for imaging analysis of co-expression of collagen genes by FISH.

Reporting for specific materials, systems and methods

Materials & experimental systems

n/a	Involved in the study	
☒	☐	Unique biological materials
☐	☒	Antibodies
☒	☐	Eukaryotic cell lines
☒	☐	Palaeontology
☐	☒	Animals and other organisms
☒	☐	Human research participants

Methods

n/a	Involved in the study	
☒	☐	ChIP-seq
☒	☐	Flow cytometry
☒	☐	MRI-based neuroimaging

Antibodies

Antibodies used	anti-Dig-POD (Roche 11633716001)<br>anti-Fitc-POD (Roche 11426346910)<br>anti-DNP-HRP (Perkin Elmer FP1129)<br>anti-muscle 6G10 (Developmental Studies Hybridoma bank 6G10-2C7)<br>anti-mouse IgG (H + L) antibody Alexa conjugated (Invitrogen A-10029, A-11031, A-21235)<br>anti-H3P (Millipore 05-817R-I, clone 63-1C-8)<br>anti-rabbit-HRP (Thermo Fisher 65-6120)

	anti-arrestin (VC1, gift from K. Agata)<br>anti-muscle V2577 (rb polyclonal antibody described in Scimone et al, 2018)
Validation	No validation of these antibodies was performed. Literature describing their common use in the planarian field is cited.

Animals and other organisms

Policy information about studies involving animals; ARRIVE guidelines recommended for reporting animal research

Laboratory animals	Asexual planarians strain CIW4 were used for all experiments and maintained under standard conditions.
Wild animals	The study did not involve wild animals.
Field-collected samples	No samples were collected from the field.
